# Curcumin Induces Apoptotic Cell Death via Inhibition of PI3-Kinase/AKT Pathway in B-Precursor Acute Lymphoblastic Leukemia

**DOI:** 10.3389/fonc.2019.00484

**Published:** 2019-06-19

**Authors:** Shilpa Kuttikrishnan, Kodappully S. Siveen, Kirti S. Prabhu, Abdul Quaiyoom Khan, Eiman I. Ahmed, Sabah Akhtar, Tayyiba A. Ali, Maysaloun Merhi, Said Dermime, Martin Steinhoff, Shahab Uddin

**Affiliations:** ^1^Translational Research Institute, Academic Health System, Hamad Medical Corporation, Doha, Qatar; ^2^National Centre for Cancer Care and Research, Hamad Medical Corporation, Doha, Qatar; ^3^Department of Dermatology Venereology, Hamad Medical Corporation, Doha, Qatar; ^4^Weill Cornell-Medicine, Doha, Qatar; ^5^Department of Dermatology, Weill Cornell University, New York, NY, United States

**Keywords:** B-Pre-ALL cells, curcumin, apoptosis, signaling, ROS

## Abstract

Acute lymphoblastic leukemia (ALL) is a significant cancer of children resulting from the clonal proliferation of lymphoid precursors with arrested maturation. Although chemotherapeutic approaches have been achieving successful remission for the majority of cases of childhood ALL, development of resistance to chemotherapy has been observed. Thus, new therapeutic approaches are required to improve patient's prognosis. Therefore, we investigated the anticancer potential of curcumin in ALL. We tested a panel of B-precursor ALL (B-Pre-ALL) cell lines with various translocations after treatment with different doses of curcumin. Curcumin suppresses the viability in a concentration-dependent manner in 697, REH, SupB15, and RS4;11 cells (doses from 0 to 80 μM). Curcumin induces apoptosis in B-Pre-ALL cell lines via activation of caspase-8 and truncation of BID. Curcumin treatment increased the ratio of Bax/Bcl-2 and resulted in a leaky mitochondrial membrane that led to the discharge of cytochrome c from the mitochondria to the cytoplasm, the activation of caspase 3 and the cleavage of PARP. Curcumin treatment of B-Pre-ALL cell lines induced a dephosphorylation of the constitutive phosphorylated AKT/PKB and a down-regulation of the expression of cIAP1, and XIAP. Moreover, curcumin mediates its anticancer activity by the generation of reactive oxygen species. Finally, the suboptimal doses of curcumin potentiated the anticancer activity of cisplatin. Altogether, these results suggest an important therapeutic role of curcumin, acting as a growth suppressor of B-Pre-ALL by apoptosis via inactivation of AKT/PKB and down-regulation of IAPs and activation of intrinsic apoptotic pathway via generation of Reactive Oxygen Species (ROS). Our interesting findings raise the possibility of considering curcumin as a potential therapeutic agent for the treatment of B-Pre-ALL.

## Introduction

Acute lymphoblastic leukemia (ALL) is a blood related human malignancy. It is commonly found in the pediatric population and is designated as B-precursor acute lymphoblastic leukemia (B-Pre-ALL). B-Pre-ALL is an aggressive hematological malignancy that frequently occurs in children (1, 2). Recently significant advances have been achieved for the treatment of pediatric ALL and outcomes for ALL in children had greatly improved. Indeed, survival rates improved continuously in 0–14 year old patients who tend to do much better than older people. In fact, survival rate for leukemia patients has been shown to reach 90% in children aged up to 14 years old while it drops to 40% in adults between 25 and 64 and it is almost 15% for those aged 65 or older (3). However, some cases of relapse due to drug resistance or toxicity are being observed (4, 5). Thus, personalized and targeted therapies are urgently needed to enhance the prognosis of ALL patients.

PI3K/AKT and its associated signaling pathways are frequently activated in B-Pre-ALL (6). Many studies suggested that PI3K/AKT plays a crucial role in oncogenesis (7, 8). PI3K/AKT activity is negatively regulated by protein tyrosine phosphatase PTEN and by SHIP which were found to be frequently mutated in many cancer cells including leukemia and lymphoma (9–12). Mutation and methylation in the PTEN gene can lead to activation of AKT (13). Activation of AKT can prevent apoptotic cell death via stimulation of anti-apoptotic signals through activation/phosphorylation of NF-kappa B, Bad, GSK3, and Forkhead (FOXO1) (14–16). An aberrantly activated PI3K/AKT pathway, therefore, offers an attractive target for chemotherapeutic agents (17, 18).

Curcumin (diferuloylmethane) is found in plant *Curcuma longa* (Linn) and has been shown to possess proapoptotic activities in various cancer cells (19–21). In animal studies, curcumin suppresses carcinogenesis of the breast, colon, liver, and skin (22–24). Curcumin induces apoptotic cell death via targeting various survival signaling pathways including inhibition of PI3-kinase/AKT, JAK/STAT3, and activation of NF-kB in many cancers (25–27). Furthermore, curcumin suppresses the expression of various antiapoptotic genes involved in the regulation of cell proliferation and apoptosis (28–30).

In this study, the antitumor activity of curcumin against B-Pre-ALL was investigated using a panel of cell lines. Curcumin suppressed cell proliferation in a dose-dependent manner via stimulation of apoptosis. Curcumin inhibited AKT and its downstream substrates molecules. Curcumin triggered intrinsic apoptotic signaling pathways by involving the interaction of cytochrome c and caspases signaling. Curcumin-mediated apoptosis is associated with the generation of reactive oxygen species. Interestingly, a combination of curcumin and cisplatin potentiated anticancer effects in B-Pre-ALL cells.

## Materials and Methods

### Reagents and Antibodies

Curcumin, CCK-8 kit, DMSO, and N-acetyl cysteine were purchased from Sigma Chemical Co (St. Louis, MO, United States) (Caspase-9, caspase-3,cleaved caspase-3,PARP,XIAP,p-Akt,Akt,GSK3,P-GSK3,FOXO1,P-FOXO1,GAPDH,cIap1,cIap2, Bcl-2, Bcl-xl, caspase 8, and cleaved caspase-8 antibodies were obtained from Cell Signaling Technologies (Beverly, MA, United States). Bax, p-H2AX, and cytochrome c antibodies and cisplatin were procured from Santa Cruz Biotechnology, Inc. (Santa Cruz, CA, United States). Annexin V fluorescein isothiocyanate (FITC), Propidium Iodide (PI), and p-H2AX (pS139) antibodies were purchased from BD Biosciences (San Jose, CA). z-VAD-FMK was obtained from Calbiochem (San Diego, CA, United States). CellROX Green was obtained from Invitrogen (MA, United States). Curcumin was dissolved in DMSO and further diluted in the cell culture media for the treatment of cells, so that the final concentration of DMSO in wells is 0.1% at the highest concentration of Curcumin used in the study. Viability assays showed that 0.1% DMSO is non-toxic to the cells (data not shown).

### Cell Culture

The 697, REH, RS4;11, and SupB15 cells were cultured and propagated described previously (31).

### Cell Viability Assay

The cell viability assay was determined in B-Pre-ALL cells in response to curcumin by using MTT assay as described previously (32).

### Annexin V FITC/Propidium Iodide Dual Staining

After curcumin treatment, RS4;11, and SupB15 cells were washed and stained with BV421-conjugated annexin-V and PI and apoptosis were analyzed by using flow cytometry as described previously (33).

### Cell Lysis and Immunoblotting

B-precursor acute lymphoblastic leukemia cells were lysed after curcumin treatment as described previously (32). Thirty to fifty micrograms of proteins were separated on SDS-PAGE, transferred to polyvinylidene difluoride (PVDF) membrane, immunoblotted using antibodies and visualized under ChemiDoc System.

### Assay for Cytochrome C Release

Cells treated with different doses of curcumin were incubated at 37°C for 24 h. After 24 h of incubation, the cells were harvested, washed, and suspended in hypotonic buffer (26). Twenty to twenty-five micrograms proteins of cytosolic and mitochondrial fractions were separated and immunoblotted with anti-cytochrome c and GAPDH.

### Measurement of Mitochondrial Membrane Potential

Cells were treated with different doses of curcumin and incubated at 37°C for 24 h. After 24 h of incubation, the cells were incubated with Muse MitoPotential working solution at 37°C for 20 min. After incubation, 5 μl of 7-aminoactinomycin D (7-AAD), was added and incubated for 5 min, and MMP was analyzed by using Muse Cell Analyzer (Merk Millipore) as described previously (34).

### Detection of DNA Damage by Comet Assay

After curcumin treatment of cells, single or double-stranded breaks in DNA were determined using Comet assay kit as per manufacturer's instructions. Briefly, after harvesting the cells, lysis was done on agarose over glass slides. Electrophoresis was carried out for 30 min, and the slides were fixed and air dried. To detect the DNA, the slides were stained with cyber green and observed under a fluorescence microscope. DNA damage can be classified based on the relative intensity and shape of the fluorescence (35).

### Measurement of Reactive Oxygen Species

B-precursor acute lymphoblastic leukemia cells were treated with curcumin and incubated for 24 h. After harvesting, the cells were washed with HBSS and stained with CellROXTM Green Reagent (Invitrogen, MA, United States). Reactive Oxygen Species (ROS) level was quantified using flow cytometry (36).

### Colony Formation Assay

The CytoSelect 96-well cell transformation assay kit (Cell Biolabs, Inc.) was used to perform the colony formation assay according to the manufacturer's protocol. Cells were treated with curcumin and 4 × 10^5^ cell/ml were seeded into a 0.4% top agar layer poured over 0.6% lower agar layer and incubated at 37°C for 8–10 days. Agar containing cells were solubilized and lysed, OD was determined at 485/530 nm. The fluorescence intensity was measured using CyQuant dye.

### Small Inference RNA Studies AKT Gene Silencing

RS4;11 cells (1 × 10^6^) were transfected with AKT siRNA (Cat no: SI02758406, Qiagen, Germany) and *Control siRNA* (Cat no: *1027281*, Qiagen) using 4D-Nucleofector™ System (Lonza). After 48 h of incubation, cells were lysed and immunoblotted with anti-AKT and other antibodies.

### Statistical Analysis

Comparisons between groups used the Student's paired *t*-test. Data are presented as the mean ± S.D. ^*^denotes as *P* ≤ 0.05 and ^**^represents as *P* ≤ 0.001 referring as the level of statistically significant.

## Results

### Curcumin Causes Dose-Dependent Cell Death in B-Pre-ALL Cells

**Figure 1:**
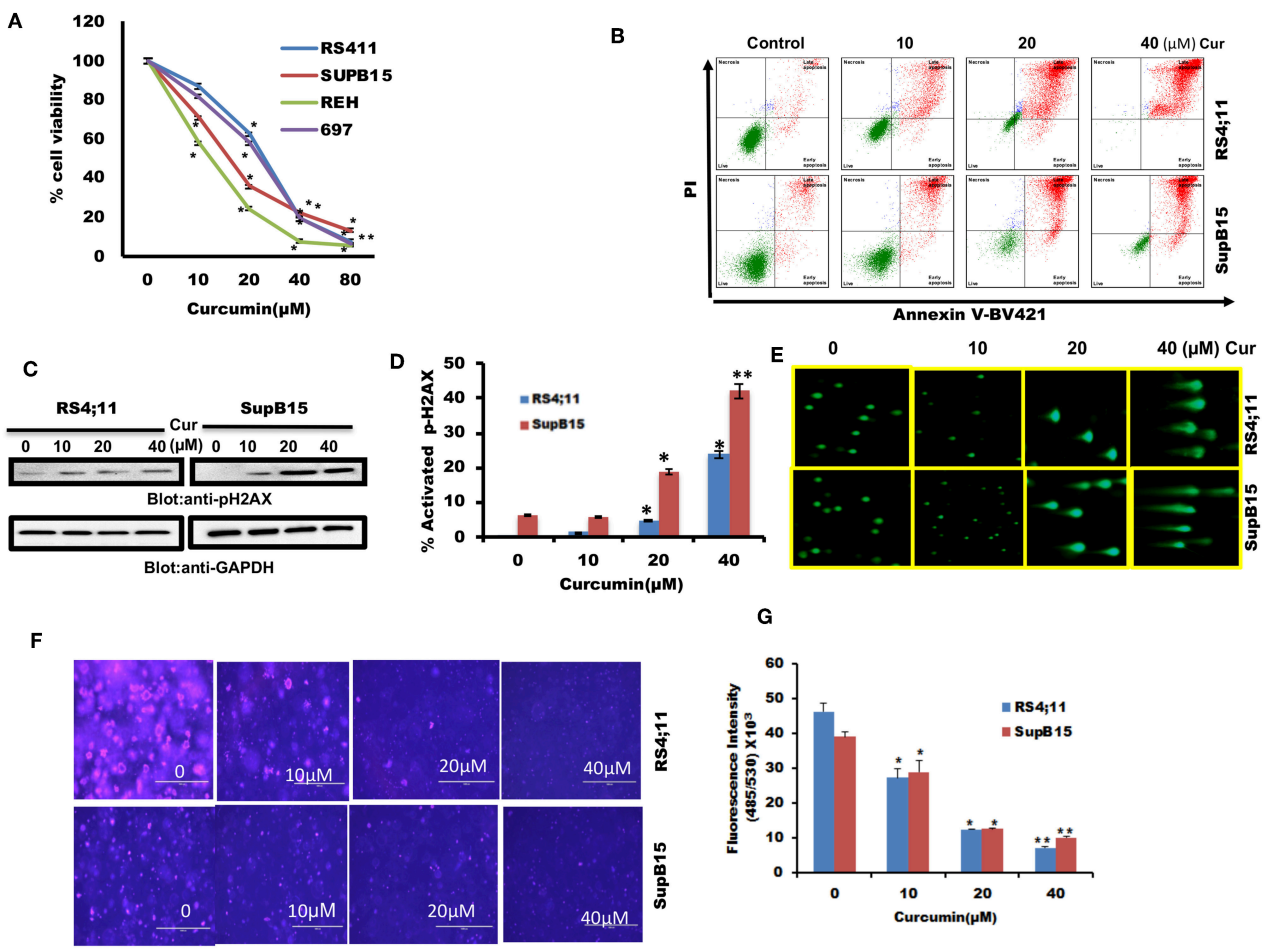
The cytotoxic effect of curcumin on Pre-ALL cells. **(A)** Curcumin inhibits the cell viability of Pre-ALL cells. RS4;11, SupB15, REH, and 697 cells were incubated with 0, 10,20, 40, and 80 μM Curcumin for 24 h. Cell proliferation assays were performed using MTT as described in Materials and Methods. The graph displays the mean ± SD (standard deviation) of three independent experiments with replicates of five wells for all the doses. *p < 0.05, **p < 0.001 **(B)** Curcumin induces apoptosis in Pre-ALL cell lines. RS4;11, SupB15 cells were treated with10, 20, and 40 μM of Curcumin for 24 h and cells were subsequently stained with fluorescein-conjugated annexin-V, PI, and subsequently analyzed by flow cytometry. **(C)** Curcumin mediated phosphorylation of H2AX in Pre-ALL cell lines. RS4;11 and SupB15 cells were treated with Curcumin as indicated in the figure for 24 h and cells were subsequently lysed and immunoblotted with anti-pH2AX antibody and GAPDH for equal loading. **(D)** Curcumin treatment induces double-stranded breaks in Pre-ALL cells. RS4;11 and SupB15 Cells were treated with 10, 20, and 40 μM of Curcumin as indicated for 24 h and cells were subsequently stained with H2AX (pS139)-Alexa Fluor 647 antibody as described in Materials and Methods and then analyzed by flow cytometry. The graph displays the mean ± SD (standard deviation) of three independent experiments for all the doses. *p < 0.05, **p < 0.001. **(E)** Curcumin-mediated DNA degradation in a single cell. RS4;11 and SupB15 cells were treated with 10, 20, and 40 μM curcumin for 24 h and cells were used to perform Comet assay to visual DNA fragmentation as described in Materials and Methods. **(F)** Effect of curcumin on the formation of colonies in B-Pre-ALL cells. RS4;11 and SupB15 cells were plated on methyl-cellulose with 10, 20, and 40 μM curcumin for 8–10 days. Colonies were stained with CyQuant dye and fluorescence intensity measured at 485/530 nm. **(G)** The graph displays the mean ± SD (standard deviation) of three independent experiments for all the doses. **p < 0.001.

We firstly determined cell viability status in response to curcumin treatment of B-Pre-ALL cells. 697, REH, RS4;11, and SupB15 cells were treated with increasing doses of curcumin and cell viability was determined by MTT reduction assay. Curcumin treatment of B-Pre-ALL cell lines inhibited survival in a dose-dependent manner (Figure 1A). A significant level of inhibition of cell viability was seen at 20 μM and above doses of curcumin in all cell lines.

#### Curcumin-Mediated Apoptosis in B-Pre-ALL Cells

We were interested in examining whether the reduced cell viability of B-Pre-ALL cells is due to apoptosis. After treatment of RS4;11 and SupB15 cells with curcumin, cells were stained with Annexin V-FITC/PI as described in Materials and Methods. As revealed in Figure 1B, curcumin treatment of B-Pre-ALL cells resulted in a dose-dependent positive Annexin V-FITC/PI staining. Phosphorylation of H2AX is a critical event for the generation of DNA damage as a consequence of double-stranded break (37). Curcumin exposure to Pre-ALL cells resulted in phosphorylation of H2AX at ser39 (Figure 1C) in both cell lines in a dose-dependent manner. Flow cytometric analysis using p-H2AX staining showed that curcumin generated a significant amount of double-strand breaks at 20 μM and above doses (Figure 1D). DNA damage was further analyzed by COMET assay after treatment of RS4;11 and SupB15 with curcumin. DNA damage in individual cells (an increase of tail) was seen in a dose-dependent manner (Figure 1E). Finally, we investigated curcumin's effect on colony formation ability of B-Pre-ALL cells. RS4;11 and SupB15 cells after treatment with curcumin were seeded on agar using CytoSelect assay kit and colonies were quantified after incubation of 8 days as described. Cells were lysed and quantified by using a fluorescence plate reader (485/520 nm). As shown in Figures 1F,G, curcumin treatment causes a dose-dependent decrease in colony formation ability of B-Pre-ALL cells. Significant inhibition of colonies was observed at 10 μM and above doses of curcumin. These data strongly support an anti-cancer potential of curcumin in B-Pre-ALL cells.

### Curcumin-Mediated Mitochondrial Apoptotic Signaling Pathway in B-Pre-ALL Cells

**Figure 2:**
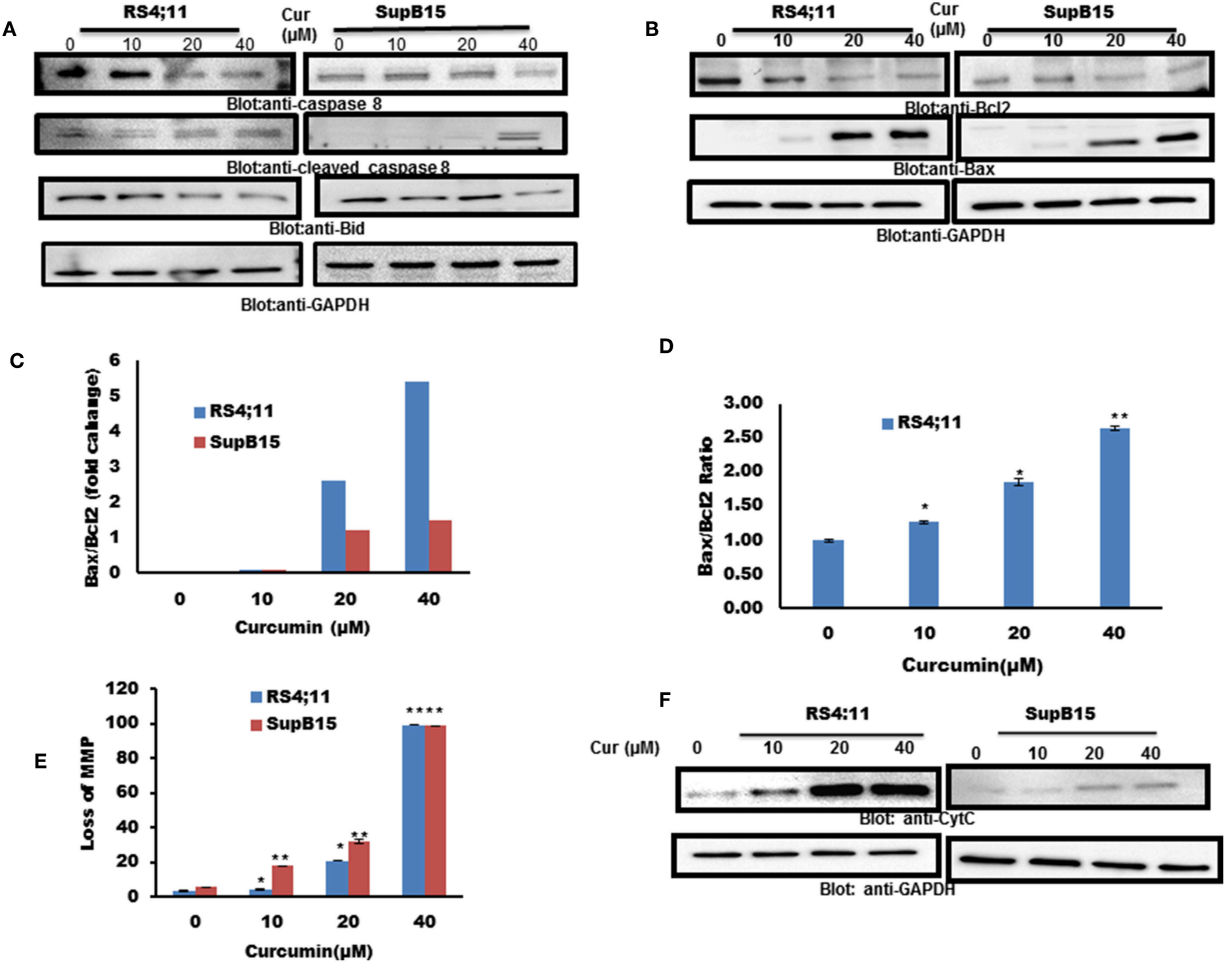
Curcumin-induced mitochondrial signaling pathways in pre-ALL cells. Curcumin induced activation of caspase-8 and BID in B-Pre-ALL. **(A)** RS4;11 and SupB15 cells were treated with increasing doses of Curcumin for 24 h as indicated. Cells were lysed and 25–50 μg of protein was separated by SDS-PAGE, transferred to PVDF membrane, and immuno-blotted with antibodies against caspase-8, BID, and GAPDH. **(B)** Effect of curcumin on Bax and Bcl-2 expression. RS4;11 and SupB15 cells were treated with increasing doses of Curcumin for 24 h as indicated. After cell lysis, equal amounts of proteins were separated by SDS–PAGE, transferred to PVDF membrane and immunoblotted with antibodies against Bax, Bcl-2, and GAPDH as indicated. Data obtained from immunoblot analyses of Bax and Bcl-2 in **(B)**. RS4;11 and SupB15 were used to evaluate effects of curcumin on Bax/Bcl-2 ratio. **(C)** Densitometric analysis of Bax and Bcl-2 bands was performed using AlphaImager Software (San Leandro, CA, United States), and data (relative density normalized to GAPDH) were plotted as Bax/Bcl-2 ratio. **(D)** RS4;11 cells are treated with and without 10, 20, and 40 μM of curcumin for 24 h and levels of Bax and Bcl-2 were determined by flow cytometry as described in Materials and Methods. The MFI values were used to calculate the Bax/Bcl-2 ratio, and the mean ± SD (standard deviation) is plotted in the graph. **p < 0.001. Curcumin treatment causes the loss of MMP in Pre-ALL cells. **(E)** RS4;11 and SupB15 cells were treated with indicated doses of Curcumin for 24 h. After JC1 staining, cells were analyzed by flow cytometry as described in Materials and Methods. The graph displays the mean ± SD of three independent of experiments. *p < 0.05 and **p < 0.001. **(F)** The curcumin-induced release of cytochrome c. RS4;11 and SupB15 cells were treated with 10, 20, and 40 μM of Curcumin for 24 h. Mitochondrial and cytoplasmic fractions were isolated as described in Materials and Methods. Cell extracts were separated on SDS-PAGE, transferred to PVDF membrane, and immunoblotted with an antibody against cytochrome c and GAPDH.

Caspase 8 can activate Bid by inducing the cleavage, to enhance apoptotic signals via the mitochondrial pathway (38). Therefore, we pursued to find out the expression of caspase-8 and Bid in response to curcumin treatment. As shown in Figure 2A, curcumin treatment resulted in reduced level of procaspase-8 with a subsequent increase in a cleaved fragment of caspase-8 indicating its activation. In addition to caspase 8, curcumin also reduced the expression of Bid protein, an indication of its cleavage (Figure 2A). Bid is a BH3-proapoptotic protein which plays a vital role in mediating Bax conformational change that leads to Bax translocation to mitochondrial membrane (39, 40). The Bcl-2 is an antiapoptotic member of Bcl-2 family, Bax, on the other hand, is a pro-apoptotic protein that plays a major regulatory function in response to a variety of upstream survival and death signals, to decide the outcome of cells (41). We, therefore, assessed the effect of curcumin action on the expression of Bax and Bcl-2 in RS4;11, and SupB15 cell line. As shown in Figure 2B, as the dosage of curcumin increased, there was an increase in Bax expression, and at the same time, Bcl-2 levels were decreased. Quantification of these protein data showed that the ratio of Bax/Bcl-2 increased with the increasing dose of curcumin (Figure 2C). The expression of Bax and Bcl-2 was also analyzed by flow cytometry using labeled Bax and Bcl-2 antibodies. Figure 2D shows that the ratio of Bax/Bcl-2 was increased with increased concentration of curcumin. The increased proportion of Bax/Bcl-2 plays a potential role in disturbing the permeabilization of mitochondrial membrane, resulting in loss of mitochondrial membrane potential (MMP). Reduced MMP level is a critical, irreversible phase for induction of intrinsic type of apoptosis (42). As shown in Figure 2E, different doses of curcumin (10–40 μM) resulted in a rapid reduction of MMP in both cell lines as determined by Muse MitoPotential Assay described in method. Cytochrome c plays a critical role in the execution of the intrinsic apoptotic pathway by activation of caspases via interaction of APAF-1 (43). As curcumin treatment of B-Pre-ALL cells reduced the MMP, this prompted us to determine the status of cytochrome c in the cytosolic fraction. The level of cytochrome c was found to be increased in both cell lines in response to curcumin treatment, strongly suggesting the involvement of intrinsic apoptotic signaling (Figure 2F).

#### Curcumin-Mediated Activation of Caspase-Cascade

**Figure 3:**
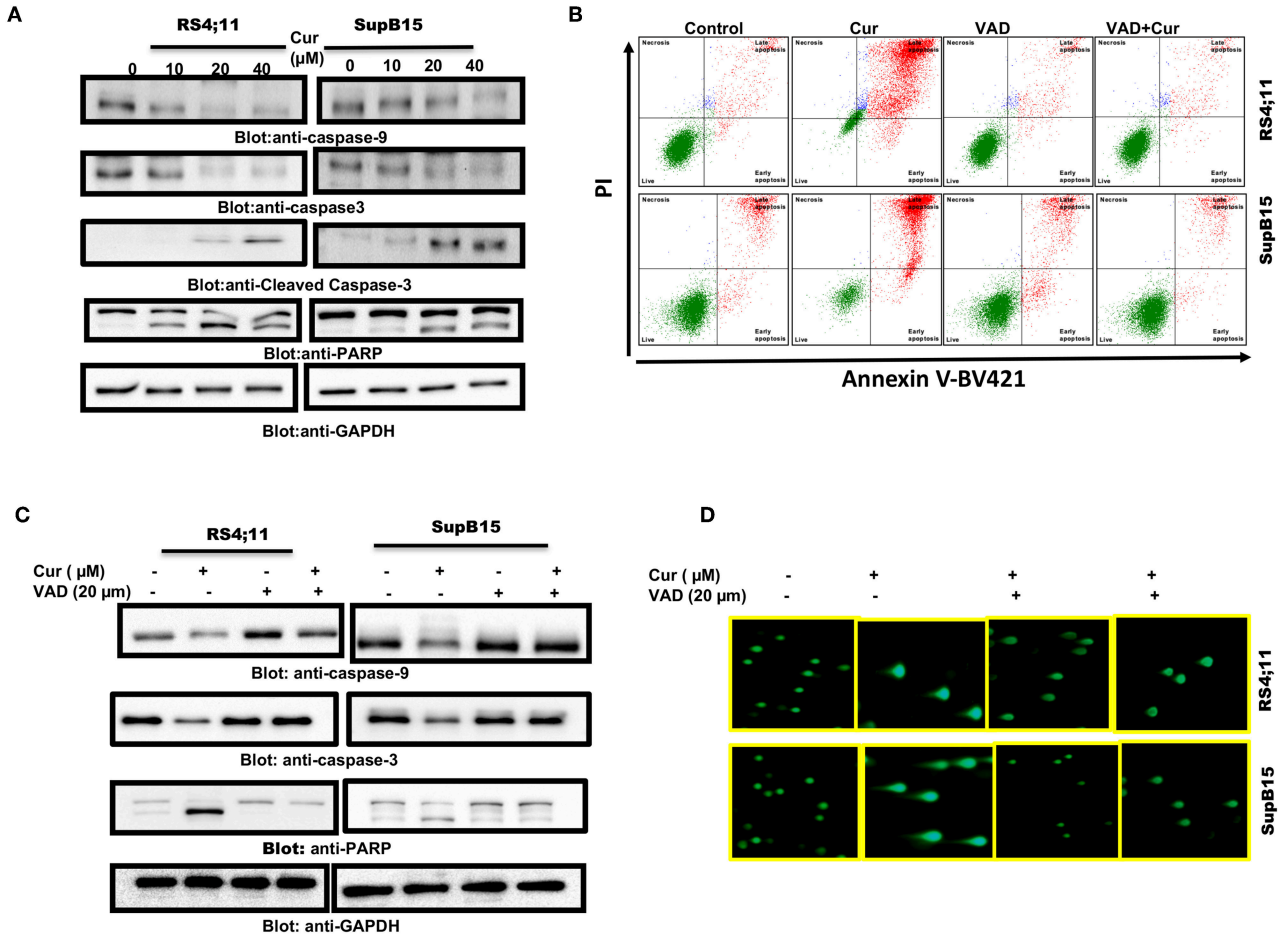
Curcumin-mediated activation of caspase cascade in Pre-ALL cells. Curcumin-induced activation of caspase-9, caspase-3, and cleavage of PARP in Pre-ALL cells. **(A)** RS4;11 and SupB15 cells were treated with and without 10, 20, and 40 μM of Curcumin for 24 h. Cells were lysed and 50 μg of proteins were separated on SDS-PAGE, transferred to PVDF membrane, and immunoblotted with antibodies against caspase-9, caspase-3, cleaved caspase-3, PARP, and GAPDH. **(B)** Effect of z-VAD-FMK on curcumin-induced apoptosis and activation of caspase-3. RS4;11, and SupB15 were pre-treated with 20 μM z-VAD-FMK for 1 h and subsequently treated with 20 μM Curcumin for 24 h. Apoptosis was measured with fluorescein-conjugated annexin-V and propidium iodide (PI) and analyzed by flow cytometry. **(C)** Pre-treatment of z-VAD-FMK prevented curcumin activated caspases. RS4;11 and SupB15 cells were treated as described above and lysed. 50 μg protein lysates were separated by SDS-PAGE, transferred to PVDF membrane, and immunoblotted with antibodies against caspase 9, pro-caspase-3, PARP, and GAPDH. **(D)** Effect of z-VAD-FMK on CURCUMIN-induced DNA damage. RS4;11 and SupB15 cells were treated as described above and induction of DNA damage was visualized using Comet assay.

Cysteine proteases are known as caspase enzymes that are involved in the execution of the programmed cell death process (44). As curcumin efficiently released cytochrome c, we, therefore, determined activation of caspase-9, caspase-3, and PARP in RS4;11, and SupB15 cells after curcumin treatment, by immunoblotting. As shown in Figure 3A, elevated levels of activated caspase-9 and caspase-3 were seen in both cell lines. PARP, an effector substrate of caspase-3, was also found to be cleaved (activated) after curcumin treatment. z-VAD-fmk an inhibitor of caspases blocked curcumin-mediated apoptosis (Figure 3B) as well as activation caspases and PARP cleavage (Figure 3C). In addition, z-VAD-fmk blocked curcumin-mediated DNA degradation (Figure 3D). These results are strongly suggesting the involvement of caspases in curcumin-mediated cell death.

### Curcumin Suppresses PI3-Kinase/AKT Pathway in B-Pre-ALL Cells

**Figure 4:**
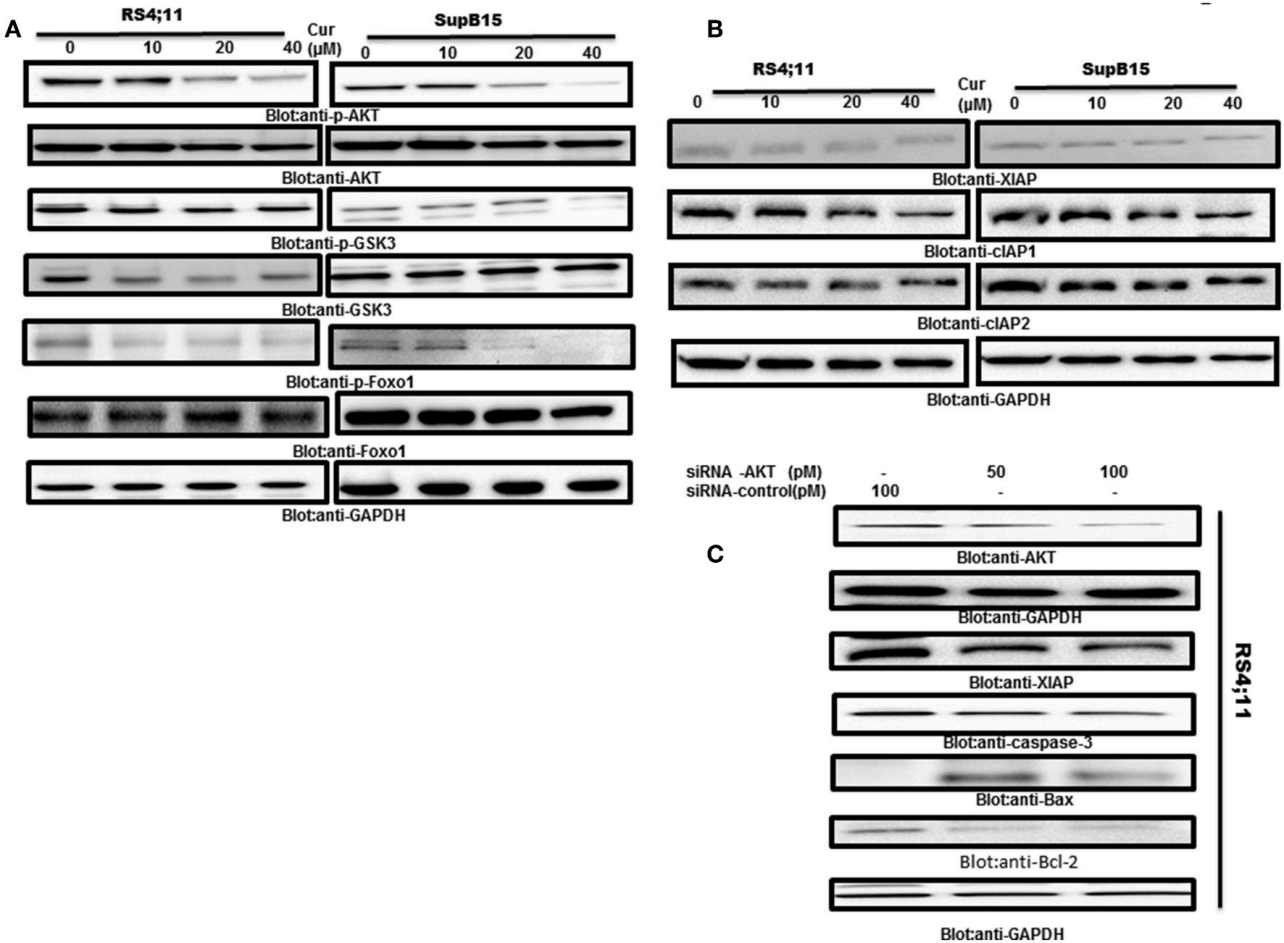
Curcumin-suppresses constitutive activated the PI3-kinase/AKT signaling pathway. **(A)** RS4;11 and SupB15 cells were treated with increasing doses of curcumin for 24 h as indicated. After cell lysis, equal amounts of proteins were separated by SDS–PAGE, transferred to PVDF membrane and immunoblotted with antibodies of p-AKT, AKT, p-GSK3, GSK3, p-FOXO1 FOXO1, and GAPDH as indicated. **(B)** Effect on curcumin on inhibitor of apoptotic proteins (cIAPs). **(B)** RS4;11 and SupB15 cells were treated with increasing doses of curcumin for 24 h as indicated. After cell lysis, equal amounts of proteins were separated by SDS–PAGE, transferred to PVDF membrane and immunoblotted with antibodies of XIAP, CIAP1, cIAP2, and GAPDH as indicated. **(C)** Gene silencing of AKT suppressed antiapoptotic proteins and induced proapoptotic proteins. RS4;11 cells were transfected with either control (100 pM) or AKT specific siRNA (50 or 100 pM). Cells extracts were separated on SDS-PAGE, transferred to PVDF membrane, and immunoblotted with antibodies against AKT, GAPDH, XIAP, caspase-3, and Bax and Bcl2.

AKT activity has been found to be inactivated by curcumin in many tumor cells (25). Therefore, we determine if AKT and its associated molecules are involved in curcumin-induced apoptosis in B-Pre-ALL cell lines. Protein lysates of RS4;11 and SupB15 cells treated with curcumin were analyzed by phosphorylated AKT antibody (p-AKTSer^473^), a marker for its activity (45). Constitutive phosphorylation of AKT was seen in RS4;11 and SupB15, cells. Interestingly curcumin treatment dephosphorylated AKT without affecting its protein level (Figure 4A). AKT mediated signaling has been shown to regulate the Forkhead family of transcription in inhibition of apoptosis (46). During the cell cycle arrest and apoptosis, theactivated FOXO1 plays an important role (47). Phosphorylation of FOXO1 by AKT leads to cell survival via inhibition of apoptosis. This prompted us to investigate whether curcumin treatment of B-Pre-ALL cells can prevent the phosphorylation of FOXO1. The basal level of constitutive phosphorylation of FOXO1 was seen in untreated RS4;11 and SupB15 cells and levels of phosphorylation were suppressed after curcumin treatment (Figure 4A). In the next experiments, we seek to determine whether suppressed GSK3, a downstream substrate of AKT which functions to help cells sustain cell survival (17). Curcumin treatment showed a weak de-phosphorylation effect on GSK3 in both cell lines (Figure 4A). These results suggest that curcumin suppressed the constitutively activated AKT signaling pathways during curcumin-induced cell death in B-Pre-ALL cell lines.

Inhibitors of apoptosis proteins (IAPs) play an essential functional role in the regulation of programmed cell death in mammalian cells (48). We, therefore, examined the effects of curcumin on the expression of IAPs in B-Pre-ALL cells. Rs4;11, and SupB15 cells were treated in the presence and absence of curcumin for 24 h. The expression level of IAPs was determined by western blotting. Curcumin suppressed the expression of XIAP and cIAP1 in a dose-dependent manner. However, there were only minimal effects on cIAP2 (Figure 4B). These results are implicating the involvement of IAP proteins in curcumin-induced apoptosis.

AKT and its associated signaling are involved in Pre-ALL cell and its targeting can lead to induction of apoptosis. We used gene silencing strategy using small interference RNA (siRNA) technology to deplete the AKT genes using AKT specific siRNA in RS4;11 cells. As shown in Figure 4C, knockdown of AKT resulted in decreased expression of XIAP and Bcl-2 antiapoptotic proteins. Interestingly, gene silencing of AKT upregulated the Bax proapoptotic protein as well-activated caspase-3, a marker of apoptosis. These results suggest that AKT is involved in curcumin-mediated apoptosis in B-Pre-ALL cells.

### Involvement of Reactive Oxygen Species (ROS) in Curcumin-Mediated Apoptosis in B-Pre-ALL Cells

**Figure 5:**
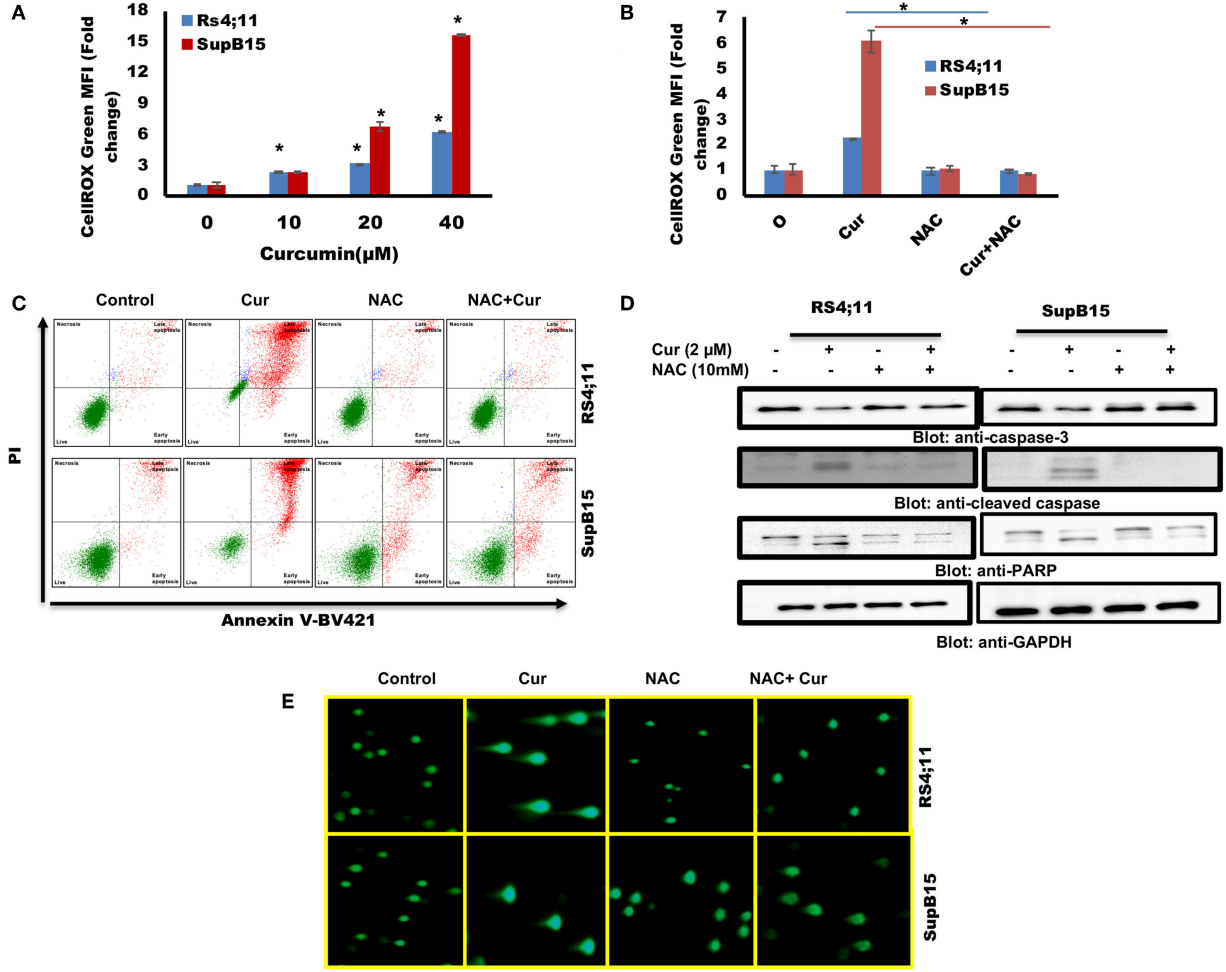
**(A)** Curcumin increases ROS generation in Pre-ALL cells. RS4;11 and SupB15 cells were treated with curcumin for 24 h level of ROS was measured by flow cytometry using CellROX Green kit as described in Materials and Methods. The graph displays the mean ± SD (standard deviation) fold change release of ROS of three experiments *p < 0.05. **(B)** Effect of NAC on the curcumin-induced generation of ROS. RS4;11 and SupB15 cells were pre-treated with 10 mMNAC, subsequently treated with 20 μM curcumin for 24 h. CellROX Green assays were performed as described in Materials and Methods. The graph displays the mean ± SD (standard deviation) fold change release of ROS of three experiments *p < 0.05. **(C)** NAC pre-treatment of pre-ALL cell prevented curcumin-mediated apoptosis. RS4;11 and SupB15 cells were pretreated with 10 mM NAC, subsequently treated with 20 μM curcumin as indicated for 24 h and apoptosis was measured by staining with fluorescein-conjugated annexin-V and propidium iodide (PI) and analyzed by flow cytometry. **(D)** NAC pre-treated pre-ALL cell prevented curcumin-mediated activation of caspases. RS4;11 and SupB15 cells were pretreated with 10 mM NAC, subsequently treated with 20 μM Curcumin as indicated for 24 h and lysed cell extracts were separated on SDS-PAGE, transferred to PVDF membrane, and immunoblotted with an antibody against procaspase 3, cleaved caspase 3, PARP, and GAPDH. **(E)** NAC pre-treated pre-ALL cell prevented curcumin-mediated DNA degradation. RS4;11 and SupB15 cells were pretreated with 10mM NAC, subsequently treated with 20 μM curcumin as indicated for 24 h cells were used to perform Comet assay to visual DNA fragmentation as described in Materials and Methods.

Involvement of ROS-induced cell death is associated with the anticancer activity of many anticancer agents (49). ROS generation in cancer cells occurs in response to many compounds (42, 50). Next, we explored the involvement of ROS in curcumin-mediated apoptosis in B-Pre-ALL cells. ROS was determined using CellROX kit after treatment of RS4;11 and SupB15 with curcumin. A dose-dependent generation of ROS was seen in both cell lines (Figure 5A). N-Acetyl Cysteine (NAC), an antioxidant that scavenges free radicals (51), blocked generation of ROS in response to curcumin (Figure 5B), suggesting the participation of ROS in curcumin-induced apoptosis.

We further investigated whether curcumin generated ROS participate in apoptotic cell death. Interestingly, curcumin-induced apoptosis was reversed in NAC pretreated cells (Figure 5C). In addition, NAC treatment also prevented the curcumin-induced activation of caspase-9, caspase-3, and PARP cleavage in response to curcumin (Figure 5D). In addition, NAC prevented curcumin-mediated DNA degradation (Figure 5E). These findings suggest that ROS play a critical role in curcumin-induced apoptosis in B-Pre-ALL cells.

### Curcumin Enhances the Therapeutic Action of Cisplatin in B-Pre-ALL

**Figure 6:**
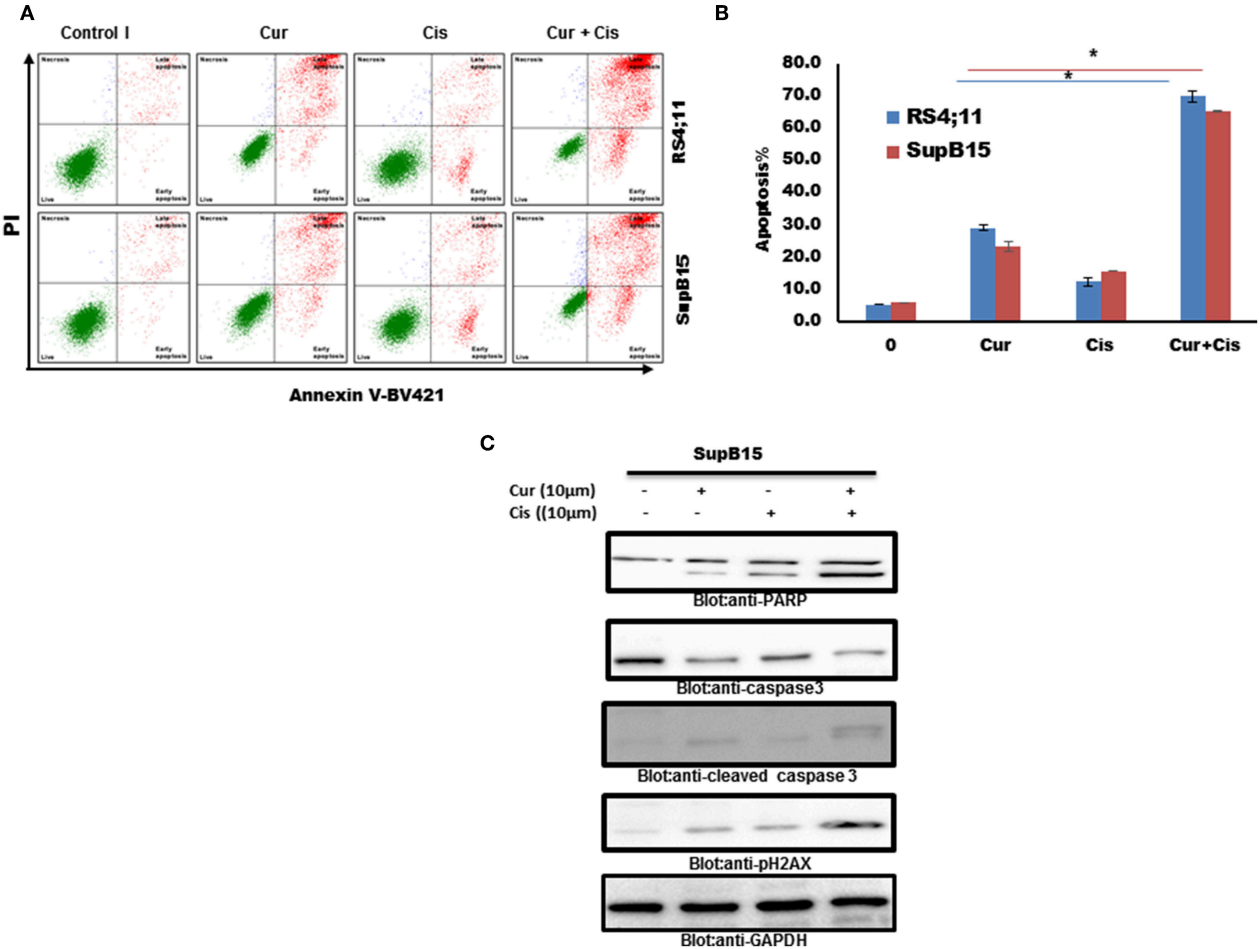
Combination treatment Pre ALL cells with curcumin and cisplatin potentiated apoptotic effects. **(A)** Curcumin and cisplatin augmented annexin-V positive cells. SupB15 cells were treated with 10 μM curcumin and 10 μM cisplatin alone or in combination for 24 h as indicated. The cells were then stained with fluorescein-conjugated annexin-V, PI, and analyzed by flow cytometry. **(B)** The graph displays the mean ± SD (standard deviation) of the percentage of apoptotic cells three independent experiments with replicates of three wells for all the doses. *p < 0.001. **(C)** SupB15 cells were treated with 10 μM curcumin and 10 μM cisplatin alone or in combination for 24 h cells were lysed and 50 μg of proteins were separated on SDS-PAGE, transferred to PVDF membrane, and immunoblotted with antibodies against caspase-3, cleaved caspase-3, PARP, p-H2AX, and GAPDH.

We tested if the combination of curcumin and cisplatin potentiated the anticancer activity better than curcumin or cisplatin alone. SupB15 cells were treated with sub-toxic doses of curcumin (10 μM) and cisplatin (10 μM) for 24 h. Apoptosis was determined by annexin staining using flow cytometry. The status of caspase activation, cleavage of PARP, and phosphorylation of p-H2AX were determined by western blotting. As shown in Figures 6A–C, the combination treatment of curcumin (10 μM) and cisplatin (10 μM) exerted stronger apoptosis than curcumin or cisplatin alone (positive Annexin staining, activation of caspases and PARP cleavage) and DNA damage (p-H2AX expression). These data suggest that curcumin potentiates the apoptotic response of cisplatin in B-Pre-ALL cells.

**Figure 7:**
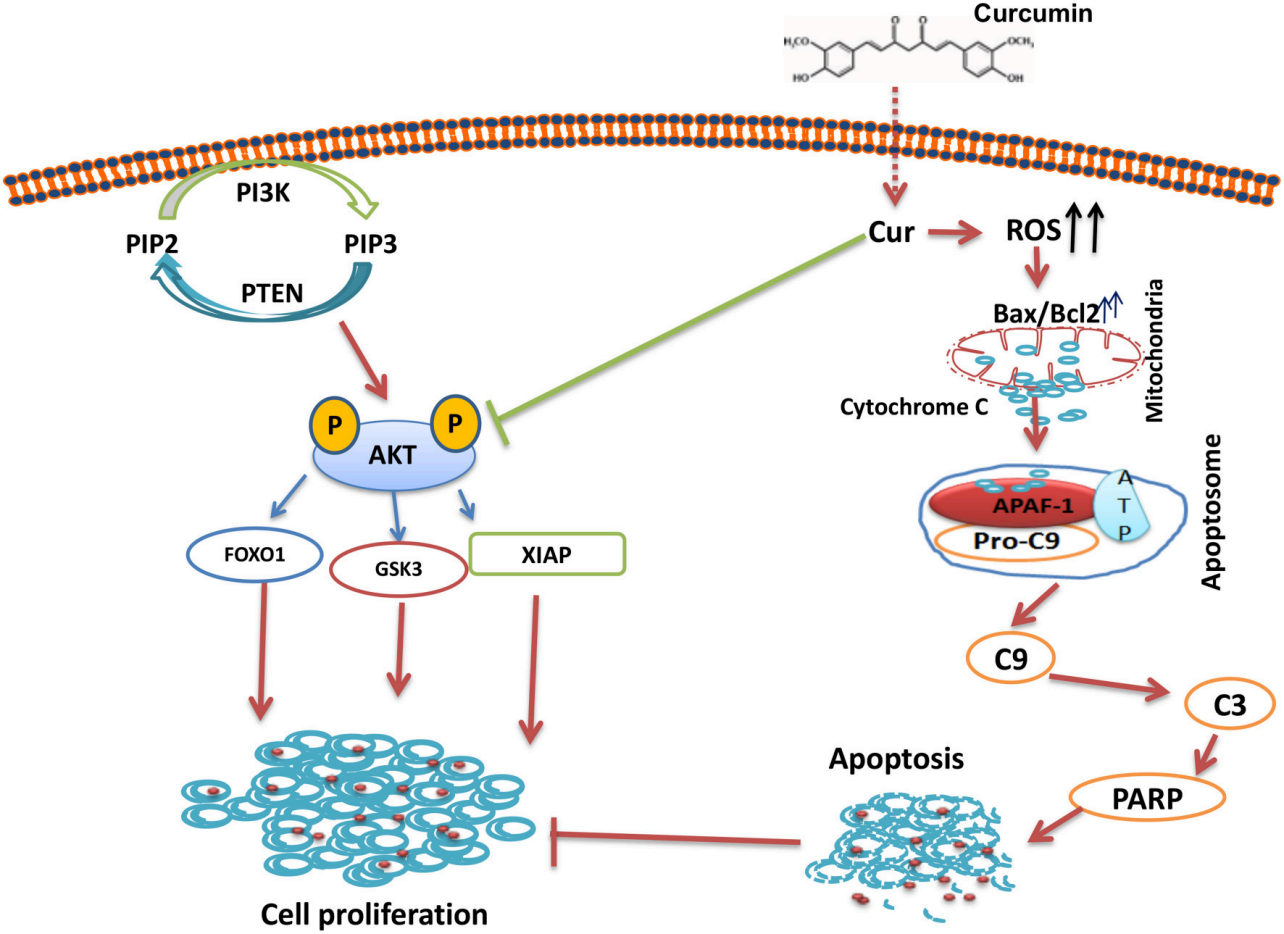
Schematic representation of curcumin mediated inhibition of cell growth via inhibition of AKT signaling and activation of mitochondrial apoptotic pathway.

In summary, our results showed that curcumin suppresses B-pre-ALL cells growth via induction of apoptosis and suppresseion of PI3-kinase/AKT signaling pathway. The Inhibition PI3-kinase/AKT activity led to the upregulation of proapototic protein Bax and the down-regulation of Bcl-2, thus, damaging the mitochondrial membrane, leading to the accumulation of cytochrome c in the cytoplasm, and the formation of apoptosome in the presence of APAF-1 and capsase-9. The Apoptosome mediated activation of caspase-cascades and cleavage of PARP lead to the generation of apoptotic cell death (Figure 7). In addition, curcumin mediates its action through the generation of ROS. Finally, curcumin can potentiate the anticancer effects of cisplatin as compared to curcumin or cisplatin alone. Taken all together, our data suggest that curcumin possesses chemopreventive/therapeutic potentials against B-pre-ALL cells.

## Discussion

The prognosis for ALL is strongly influenced by the age at diagnosis, with lower survival described in adult population. In general, around 70% of people with ALL will survive for 5 years or more after they are diagnosed. Outcomes for ALL in children had greatly improved over the second half of the twentieth century. Indeed, survival rates improved continuously in 0–14 year old patients who tend to do much better than older people. In fact, survival rate for leukemia patients has been shown to reach 90% in children aged up to 14 years old while it drops to 40% in adults between 25 and 64 and it is almost 15% for those aged 65 or older (3). Even though advancement has been made for the treatment of children ALL, cases of relapse are still observed due to drug resistance or toxicity (4, 5). In this study, we studied the anticancer activities of curcumin, a plant-derived compound using a panel of B-Pre-ALL cells. Curcumin strongly inhibited the survival of these cells via induction of apoptosis. Curcumin mediated cytotoxic effect has been shown in B-Pre-ALL via apoptosis (52). There are two major apoptotic processes; intrinsic apoptotic cell death where mitochondrial signaling plays a vital role in the execution of cell death (53). The other type of apoptosis is known as receptor-mediated cell death where death receptors are involved in the killing of the cell (53). Most of the anticancer agents affect mitochondrial signaling as well as activation of caspases (54). Our data showed that the expression of antiapoptotic protein Bcl-2 reduced in curcumin-treated cells with concomitant increased of Bax expression. An increase of Bax/Bcl-2 ratio in response to curcumin in B-Pre-ALL cells led to the formation of mitochondrial pores, an event that can result in disruption of mitochondrial membrane leading to accumulation of cytochrome c in the cytoplasm (55). Curcumin mediated cytochrome c secretion in cytoplasm then led to activation of caspase signaling and cleavage of PARP. Furthermore, a broad-spectrum of caspase inhibitors efficiently abrogated curcumin-induced caspase-mediated apoptosis. These findings strongly propose that activation of caspases is involved in curcumin-induced cell death.

Dysregulated signaling pathways that are in governing the growth and apoptosis of cancer cells can be used as a potential target for chemopreventive agents. We investigated the involvement of PI3-kinase/AKT signaling pathways in curcumin-mediated apoptosis. PI3K/AKT/mTOR signaling pathway is found to be activated in B-Pre-ALL (6). Aberrantly activated survival signaling pathways have been frequently observed in many tumor cells including hematological malignancies (6, 56). PI3-kinase/AKT signaling pathway plays a critical role in the sustained growth and survival of tumor cells. PI3-kinase/AKT pathway is aberrantly activated in various cancers (57). Recently, Simioni et al. (58) have demonstrated that inhibition of mTOR/AKT signaling by Torin-2 suppresses the feedback activation of PI3K/AKT in B-Pre-ALL cells. Our data showed that B-Pre-ALL cells express p-AKT and its associated signaling pathways including GSK3 and FOXO1. Curcumin dephosphorylated AKT, GSK3, and FOXO1 in a dose-dependent manner in B-Pre-ALL cells cell lines. Activation of PI3-kinase/AKT pathway also plays a significant role in the regulation of antiapoptotic proteins such as Bcl-Xl, XIAP, and cIAPs (59). Curcumin suppressed the expression of these survival proteins along with inactivation of AKT pathway. Genetic knockdown of AKT with siRNA resulted in suppressed PI3-kinase/AKT pathway along with the expression of antiapoptotic genes XIAP and Bcl-2. In addition, gene silencing of AKT also upregulated proapoptotic genes Bax and activated caspase 3 suggesting that AKT signaling is involved in curcumin-mediated apoptosis in B-Pre-ALL cells.

Reactive Oxygen Species generation by anticancer agents can prime cancer cells to cytotoxic death as these cells have a depleted level of anti-oxidants (60). Our data showed that curcumin generated ROS in a dose-dependent manner in B-Pre-ALL cells. NAC, a scavenger of ROS efficiently blocked curcumin-mediated activation of caspases and apoptosis. Normal and tumor cells have different levels of susceptibility to survive the deleterious effect of ROS. High levels of ROS can induce cell death. In normal cells, the generation of ROS is controlled due to a high level of antioxidant; therefore the level never reached the death threshold (60). In contrary, the cancer cells produce ROS and have depleted level of antioxidants. Therefore, any compound/drug that can increase the level of ROS can induce the death of the cancer cells because of reaching the death threshold (60). Curcumin-mediated apoptosis is in concordance with these findings and our data strongly suggest that ROS is involved in the curcumin-mediated anticancer activity.

Curcumin significantly enhances cisplatin-mediated apoptotic cell death as compared to curcumin or cisplatin alone in B-Pre-ALL cells. In addition, this combination of drugs was able to induce a robust activation of caspase-3 and PARP cleavage. Combination treatment of SupB15 cell line with sub-toxic doses of curcumin and cisplatin enhances the generation of double-strand breaks as indicated by phosphorylated H2AX.

In conclusion, our results show that curcumin suppresses B-pre-ALL cells growth and proliferation by inactivation of the PI3-kinase/AKT signaling pathway. Inhibition of PI3-kinase/AKT activity leads to the activation of apoptosis via the down-regulation of antiapoptotic proteins including Bcl-2, and XIAP. Curcumin mediates its action through the generation of ROS. Finally, curcumin can potentiate the anticancer effects of cisplatin as compared to curcumin or cisplatin alone. Taken altogether, our data suggest that curcumin possesses the chemopreventive/therapeutic potentials against these B-pre-ALL cells.

## Author Contributions

SK and KS performed the experiment and helped in experiment designing, data analysis, and manuscript writing. KP, AK, EA, TA, and SA performed the experiment and data analysis. MM, SD, and MS helped with manuscript writing, editing, and proofreading. SU contributed to experiment design, data analysis and manuscript writing and proofreading.

### Conflict of Interest Statement

The authors declare that the research was conducted in the absence of any commercial or financial relationships that could be construed as a potential conflict of interest.
